# Pediatric Cranial Dog Bite Injuries: More than Meets the Eye

**DOI:** 10.5811/cpcem.2022.4.56178

**Published:** 2022-08-06

**Authors:** Shanze A. Tahir, Karen Z. Carver, Alex Cappitelli, Lissa Baird, Ashley Marchese, Ingrid M. Ganske

**Affiliations:** * Harvard Medical School, Boston, Massachusetts; † Harvard Medical School, Boston Children’s Hospital, Department of Plastic and Oral Surgery, Boston, Massachusetts; ‡ Harvard Medical School, Boston Children’s Hospital, Department of Neurosurgery, Boston, Massachusetts; § Harvard Medical School, Boston Children’s Hospital, Department of Emergency Medicine, Boston, Massachusetts

**Keywords:** trauma, imaging, neurosurgery, plastic surgery

## Abstract

**Case Presentation:**

A six-month-old female presented to a community hospital with small lacerations to the scalp, face, and left eyelid from a dog bite injury. Computed tomography imaging revealed an impacted right frontal bone fracture and left superior orbital fracture, prompting transfer, neurosurgical repair, and infectious disease management of the injury.

**Discussion:**

This report highlights the importance of having a high level of suspicion for deeper injury in pediatric and especially infant craniofacial dog bites, obtaining radiographic imaging, and initiating appropriate multidisciplinary triage to prevent life-threatening infection and complications.

## CASE PRESENTATION

A six-month-old female presented to a community hospital with superficial-appearing lacerations of the scalp, face, and left eyelid after a dog bite. The dog and infant were both up to date on immunizations. Upon presentation, the patient was alert and stable. Computed tomography imaging demonstrated right frontal impacted calvarial fracture with displaced fragment and adjacent right frontal intraparenchymal hemorrhagic contusions, as well as a left superior orbital fracture (Image). The patient was treated with ampicillin-sulbactam and transferred in stable condition to a tertiary care center for neurosurgical intervention and management.

Upon transfer, the patient was neurologically intact. The infectious disease (ID) team recommended treatment with ceftriaxone and metronidazole for central nervous system coverage and broad-spectrum coverage of common canine oral flora, including *Pasteurella canis*, *Viridans streptococci*, *Staphylococcus aureus*, and *Fusobacterium*. Neurosurgery took the patient to the operating room for irrigation and debridement and elevated and repaired the depressed skull fracture, contouring the calvarium to its normal frontal shape. Plastic surgery repaired the remaining facial and eyelid lacerations.

Following repair, the patient was admitted to the intensive care unit for monitoring for two nights and ultimately discharged on postoperative day five. According to ID recommendations, the patient was continued on ceftriaxone and metronidazole for four weeks post-surgery. The patient recovered without seizures, emesis, lethargy, fever, or weakness. Follow-up magnetic resonance imaging four months post injury showed evolving right frontal lobe encephalomalacia at the site of presentation of hemorrhagic contusions but no evidence of intracranial abscess, fluid collection, or parenchymal edema to suggest ongoing infection. The lacerations healed well.

## DISCUSSION

Although there are a handful of reports in the craniofacial and neurosurgical literature^1^–^4^ of severe intracranial injury from dog bites, emergency medicine diagnosis and triaging of such injuries has not been well reported.^5^ This case highlights the important role of imaging in the emergency evaluation of dog-bite injuries to the head in infants to specifically assess for occult skull fracture, even when lacerations may appear superficial. The thin infant cranium is more easily injured, and greenstick fractures may be difficult to palpate. Using radiographic imaging to evaluate the extent of the penetrating injury can help direct prompt triage, consultations, and management to minimize the risk of life-threatening complications from untreated deep infection.

Open skull fractures and depressed skull fractures with underlying mass effect or aesthetic implications are both indications for neurosurgical washout and repair. Our standard management of such fractures entails prophylactic pre- and intraoperative antibiotics, as well as postoperative antibiotics for polymicrobial exposure. Whereas punctate bite wounds in soft tissue alone are often left open to drain and heal secondarily, such injuries over open skull fractures are typically closed after thorough irrigation, debridement, and fracture reduction. Follow-up imaging is obtained to rule out abscess development.

**CPC-EM Capsule**

What do we already know about this clinical entity?

*Infants are more susceptible to skull fractures from dog bite lacerations to the head*.

What is the major impact of the image(s)?

*Computed tomography imaging showed a depressed skull fracture under the superficial-appearing puncture wound. This finding necessitated surgical intervention*.

How might this improve emergency medicine practice?

*Beware of superficial-appearing scalp or facial dog-bite injuries in infants; imaging may reveal skull injuries that portend risk of life-threatening complications*.

We recommend that clinicians be wary of minor-appearing dog bite lacerations of the scalp in infants and strongly suggest obtaining imaging in the emergency department for all young patients with dog bites to the head.

## Figures and Tables

**Image:**
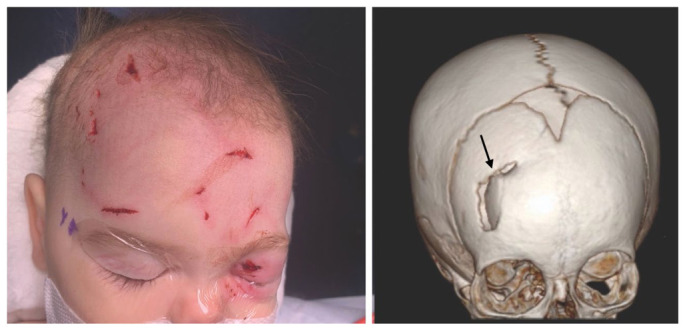
(Left) Facial and scalp lacerations; (Right) Computed tomography reconstruction demonstrating right frontal depressed skull fracture (arrow) under small scalp puncture wound from a dog bite.
